# Treatment of Constipation and Diarrhœa

**Published:** 1876-04

**Authors:** 


TREATMENT OF CONSTIPATION AND DIARRHŒA.—The New York *Medical Journal* quotes from the *Gaz. Med. di Roma:*

"Every one knows how difficult defecation sometimes is for those persons who are afflicted with habitual constipation. In such cases relief is often sought from injections of cold water, with the occasional addition of oil or soap. The following clyster is recommended as infallible in such cases:

"Take a tumbler and fill it half-full of water at the temperature of the room, pour in a few drops of tincture of camphor, just enough to give the water a slight sapidity, then fill the glass with water. Inject this slowly into the rectum till about sixty or eighty grammes have been introduced. At first no effect is perceived, but in about ten minutes the desire to defecate becomes irresistible. The effect becomes energetic in proportion to the quantity of tincture of camphor added. After the defecation it is well to repeat the injection of a small quantity of the same mixture and retain it in the rectum, which can readily be done, so as to prevent constipation on the following day.

"Although at first sight it may seem rather improbable, the same remedy is also useful in checking diarrhœa, even though it may have been persistent. The injection should be extremely slow; after the dejection it should be repeated two or three times. The quantity of tincture should also be increased."

